# Bibliography

**Published:** 1904-07-15

**Authors:** 


### BIBLIOGRAPHY.

### ESSAY ON IRREGULARITIES OF THE TEETH.

BY J. SIM WALLACE, D. SC., M.D, L.D.S.

Published by the Dental Manufacturing Company, London.

This little volume is not without merit, though if its teachings were to be adopted as a whole the evil would outweigh the good. The importance of the proper performance of the functions of the jaws and teeth is considered at some length, and while some might differ with Mr. Wallace in regard to just how this brings about the desired development, no one, I think, doubts that exercise of the jaws and tongue are essential to their proper growth. The author puts forth the theory that the tongue is the dominating influence which has to do with the size of the jaws, or more particularly with the size of the dental arches, and that its proper development requires more vigorous exercise than is demanded in the mastication of "civilized" foods. He argues that functional activities is not so essential to the development of the bone tissues as to muscle, hence, he concludes that generous mastication hastens the growth of the jaws by causing an enlargement of the tongue, and that this in turn is the stimulus producing the necessary enlargement of the jaws for the accommodation of the permanent teeth.

His discussion of this subject is interesting reading, and no matter whether his theory of the modus operandi is entirely correct or not, the fact remains that there is no escape from the evils that follow disuse of the organs of mastication. The important part played by the tongue in certain types of mal-occlusion has been largely overlooked, no doubt, and this little work is of value in bringing this phase of the etiology of mal-occlusion of the teeth forcibly to our attention. It is also pleasing to note that pathological conditions in the nasal and post-nasal regions are given at least a small measure of the attention that they deserve.

The chapters devoted to "Prevention of Irregularities" are good so far as they go; but their value would be enhanced by devoting some consideration to lack of dental attention and also to the evils following unwise dental operations. No text-book could do its full duty when its chapters on "prevention" do not specifically deal with the deplorable results following the extraction of the first permanent molars.

In the comparatively few pages devoted to treatment the author shows a lack of familiarity with modern operative methods which is deplorable. He goes on to say that there are four distinct objects of treatment, namely, to improve the appearance; to improve mastication and articulation; to prevent caries; and to leave the teeth at the end of the treatment in a state of equilibrium. A few pages farther on he says that to establish these conditions there are four factors to be borne in mind:

1. The retention of each tooth by its approximal teeth.

2. The retention of each tooth by the occluding teeth.

3. The retention of each tooth by the tongue on one side.

4. By the lips and cheeks on the other. Notwithstanding this good advice he advocates the extraction of first bicuspids in cases where the molars have for any reason taken up a position too far forward in the jaw, and ignores the very first condition which he mentions as being essential to the permanent retention of the teeth, by saying that even though a diastema results from this that it is of little consequence. Close observation leads the reviewer to believe that this is bad advice, for oftener, than otherwise, it will be followed by a tipping forward of the molars and bicuspids or a settling back of the incisors and cuspids and in either case the interproximal spaces are destroyed, to a degree at least, and the masticating capacity of the teeth is also injured. By modern methods of treatment it is possible to move the molars distally and thus avoid the need for extracting.

Where, because of destruction by caries, it becomes necessary to extract a permanent molar, the author advocates the removal of the corresponding upper or lower molar and suggests an utterly impracticable method of closing these spaces. When the association of rhinological disturbances and mal-occlusion of the teeth is more generally understood, men will hesitate more than ever to extract a permanent upper tooth before the complete development of the face, for, by so doing, they would unquestionably interfere with the normal development of the nasal passages. The book would be a safer one in the hands of students if the chapters on treatment were eliminated entirely.

